# Transcriptional Patterns of Lower-Grade Glioma Patients with Distinct Ferroptosis Levels, Immunotherapy Response, and Temozolomide Sensitivity

**DOI:** 10.1155/2022/9408886

**Published:** 2022-05-10

**Authors:** Zewei Tu, Jingying Li, Xiaoyan Long, Lei Wu, Xingen Zhu, Kai Huang

**Affiliations:** ^1^Department of Neurosurgery, The Second Affiliated Hospital of Nanchang University, Nanchang, Jiangxi 330006, China; ^2^Jiangxi Key Laboratory of Neurological Tumors and Cerebrovascular Diseases, Nanchang, Jiangxi 330006, China; ^3^Institute of Neuroscience, Nanchang University, Nanchang, Jiangxi 330006, China; ^4^Department of Comprehensive Intensive Care Unit, The Second Affiliated Hospital of Nanchang University, Nanchang, China; ^5^East China Institute of Digital Medical Engineering, Shangrao, China

## Abstract

**Background:**

Many studies have defined a critical role for ferroptosis in cancer progression and therapy, but it is unclear how ferroptosis regulates tumor immunity or tumor microenvironment (TME).

**Methods:**

In this study, 24 ferroptosis-regulators were assessed by nonnegative matrix factorization (NMF) consensus clustering to identify ferroptosis patterns in lower-grade gliomas (LGGs). Cell-type Identification By Estimating Relative Subsets Of RNA Transcripts (CIBERSORT) method and single sample gene set enrichment analysis (ssGSEA) were used to quantify immune cell infiltrations. The PCA algorithm was used to develop the ferroptosis-related score (FRscore) to measure ferroptosis levels.

**Results:**

Two LGG subgroups named ferroptosis-related clusters 1 (FRC1) and 2 (FRC2), with distinct ferroptosis levels, immune infiltrations, and clinical outcomes were determined in 1,407 LGG samples. A well-designed scoring system was developed to evaluate the ferroptosis levels in LGG patients based on the FRSig gene profile and divided patients into low- and high-FRscore subgroups. Patients with low FRscores had lower ferroptosis levels and prolonged survival time and were expected to benefit from immune checkpoint blockade (ICB) therapy and showed higher sensitivity to TMZ chemotherapy. Findings also showed that the PI3K-AKT-mTOR pathway is activated by ferroptosis induction in SW1088 cells.

**Conclusions:**

This study highlights the critical role of ferroptosis in TME formation and shaping, and quantitatively assessing ferroptosis levels in individual tumors can help to define the intratumor microenvironment and formulate precise treatment strategies for LGG patients.

## 1. Introduction

Ferroptosis is a newly defined type of programmed cell death (PCD), different from apoptosis and autophagy, that is characterized by iron-dependent lipid hydro-peroxide accumulation [1]. Sufficient intracellular iron is needed for ferroptosis, and blockade of X_C_^−^ system (a cystine/glutamate antiporter system) and inhibition of glutathione peroxidase 4 (GPX4) can break the redox balance and increase lipid peroxidation, ultimately resulting in ferroptosis [2–4]. Recent studies have focused on the role of ferroptosis in the tumor microenvironment (TME) and shown that it plays a vital but dual function in cancer promotion and suppression. For example, ferroptosis is induced by erastin, a well-known ferroptosis-inducer molecule, and impairs the growth vitality of colorectal cancer cells [5]; however, erastin-induced glioblastoma (GBM) cells show increased migration, and TMZ-resistant GBM cells are more sensitive to ferroptosis [6]. In addition, research has shown that molecules induced by ferroptosis can effectively kill pancreatic, liver, and kidney cancer cells [3, 7–9]. In a previous study, Liu et al. analyzed the role of FRGs in pan-cancer and designed a ferroptosis potential index (FPI) to evaluate ferroptosis trends, helping subsequent research to better understand the role of ferroptosis and its regulators in various cancers [10]. However, the prognostic role of FPI and FRGs in lower-grade gliomas requires further investigation.

Lower-grade glioma (LGG), comprised of WHO grade II and III gliomas, is a subgroup of intracranial tumors that are diffuse-invasive, recrudescent, and drug-fast [11, 12]. LGG accounts for approximately 29% tumors in the central nerve system (CNS), and the median survival time of LGG patients is about 7 years, which is longer than the 5-year survival time of GBM patients [13–15]. Surgical resection along with chemotherapy and/or radiotherapy is the general treatment regimen; however, high recurrence and drug-resistance ratios or progression to GBM complicates conventional therapy. Studies show that glioma stem cells (GSC) cause LGG to be unresectable and that distinctions in the immune microenvironment of LGG may change treatment responsiveness [16].

Gliomas have an immune-suppressive nature which shapes protumor immunity and dampens the response to treatment [14, 17, 18]. This may be due, in part, to increase expression of immunosuppressive factors, like programmed cell death 1 ligand (PD-L1), interleukin 10 (IL-10), indolamine 2,3-dioxygenase (IDO), and transforming-growth factor *β* (TGF-*β*), in the glioma microenvironment [19–22]. In addition, immune checkpoint inhibitors (ICIs) have had remarkable therapeutic effects in various cancers, but their application in gliomas has been postponed due to difficulties in bypassing the blood-brain barrier (BBB) [23, 24]. The special immune microenvironment of intracranial glioma has made immunotherapy challenging. Improving knowledge of the glioma immune-suppressive microenvironment will aid in developing effective glioma-specific immunotherapies [17].

Due to intraglioma heterogeneity, individual and detailed therapeutic regimens have been formulated based on newer molecular characteristics that might have clinical benefit for LGG patients [25–27]. While ferroptosis is not fully understood, this method of cell death plays an important role in various cancers, and studies indicate that ferroptosis may be a good target for new therapies [4]. In the study presented here, the effects of FRGs and FPI in the prognosis and tumor microenvironment of LGGs were analyzed by integrating the transcriptomic and genomic information of 1,407 LGG samples from the Cancer Genomic Atlas (TCGA), Chinese Glioma Genomic Atlas (CGGA), and Gene Expression Omnibus (GEO) datasets. By the clustering of nonnegative matrix factorization (NMF) of 24 FRGs, two ferroptosis clusters in LGGs with distinct immune status and clinical prognosis were identified. Moreover, individual scoring was developed to reflect ferroptosis level, prognosis, ICI response, and TMZ sensitivity of LGG patients. Findings also showed that the PI3K-AKT-mTOR pathway was activated by ferroptosis stimulation *in vitro*. These results support an important role for ferroptosis in the LGG microenvironment and during resistance to chemotherapy and suggest a potential function for the PI3K-AKT-mTOR pathway in glioma cell survival following ferroptosis induction.

## 2. Methods and Materials

### 2.1. Public Dataset Acquisition and Preprocessing

Postoperation LGG patients survived longer than 1 month along with full transcription data were included; totally, 1407 LGG patients were enrolled for subsequent bioinformatic and statistical analysis, including those from TCGA-LGG (*n* = 477), CGGA-mRNA_seq325 (*n* = 170), CGGA-mRNA_seq693 (*n* = 379), GSE16011 (*n* = 103), GSE61374 (*n* = 137), and Rembrandt (GSE108474, *n* = 141). RNA-seq data of TCGA dataset were downloaded from UCSC Xena website (https://xenabrowser.net/), and FPKM (fragments per kilobase per million) format data were transformed into TPM format (transcripts per kilobase million). Corresponding clinicopathological information of the TCGA cohort was obtained from cBioPortal website (https://www.cbioportal.org/). The mRNA expression data and relative clinical data of the two CGGA RNA-seq cohorts were curated from the CGGA website (http://www.cgga.org.cn/download.jsp). For three microarray data cohorts from GEO repository, we downloaded the raw data of “CEL” files from GEO repository (Gene Expression Omnibus, https://www.ncbi.nlm.nih.gov/gds) and processed them with a robust multiarray averaging (RMA) method to achieve background adjustment and quantile normalization using the R packages “affy” [28] and “simpleaffy” [29]. Batch effects existing between CGGA-RNA-seq cohorts or among GEO microarray cohorts were adjusted by the “ComBat” function of “sva” [30] R package, and we got two syncretic LGG cohorts (meta-CGGA and meta-GEO) in this way. Matched genomic mutation information, including single nucleotide polymorphism (SNP) data and copy number variation (CNV) data, of TCGA-LGG was obtained from the UCSC Xena. The clinicopathological features of included LGGs are summarized in Table 1.

### 2.2. Nonnegative Matrix Factorization (NMF) Clustering of LGGs

We retrieved the 24 ferroptosis regulator genes from the earlier publications, and the expression matrixes of the 24 FRGs were extracted from the three independent LGG cohorts, respectively, for subsequent unsupervised NMF clustering analysis. We performed NMF clustering using the “NMF” R package (version 0.23.0) [31] on the TCGA-LGG cohort and the two meta LGG cohorts. The parameters of “brunet” method and 100 nruns were used to implement unsupervised NMF clustering, and optimal cluster number *k* was determined on the results of cophenetic, dispersion coefficients.

### 2.3. Gene Set Variation Analysis (GSVA) and Well-Defined Biological Process Signatures

We retrieved the gene list of Hallmark gene sets from the MSigDB database (https://www.gsea-msigdb.org/gsea/msigdb/, v7.4) to quantify the biological states or processes of each LGG sample by using GSVA algorithm [32]. Mariathasan et al. already established a series of well-defined gene signatures related with some typical and crucial biological processes like immune-checkpoint, antigen processing machinery (APM), CD8 T-effector, epithelial-mesenchymal transition markers (EMT1/2/3), angiogenesis, pan-fibroblast TGF-*β* response signature (Pan-F-TBRS), WNT targets, DNA damage repair, mismatch repair, nucleotide excision repair, DNA replication, cell cycle, cell cycle regulators, Fanconi anemia, homologous recombination, FGFR-related genes, and KEGG discovered histones, and we scored each signature for LGGs using the method defined in earlier research [33]. The KEGG pathway analysis for the upregulated genes in FRC2 was determined by the R package “clusterProfiler” [34].

### 2.4. Ferroptosis Potential Index (FPI) Calculation

The ferroptosis potential index (FPI) was quantified to represent the ferroptosis levels or trends of LGG samples according to a previous study [10]. The FPI was constructed based on the ssGSEA method using the gene expression data of ferroptosis core positive components (PCs) of ACSL4, ALOX15, GPX4, LPCAT3, NFE2L2, NCOA4, NOX1, NOX3, NOX4, NOX5, SLC3A2, and SLC7A11 and negative components (NCs) of FDFT1, HMGCR, COQ10A, and COQ10B.

### 2.5. Tumor Immune Infiltration Evaluation by ssGSEA Algorithm and CIBERSORT

The infiltrated abundance of 28 types of immune cells in the LGG microenvironment was evaluated by single sample gene set enrichment analysis (ssGSEA). Relative characteristic gene sets for quantifying immune cell abundance were retrieved from a previous publication [35], and enrichment scores computing by ssGSEA algorithm were utilized to represent the infiltration levels of the 28 immune cell types in each LGG sample [36]. Besides, the CIBERSORT method [37], a kind of deconvolution algorithm to quantify immune cell infiltration levels, was also utilized to evaluate the infiltration abundance of 22 diverse immune cells using relative mRNA expression profile of LGG patients.

### 2.6. Recognition of Differential Expressed Genes (DEGs) between Different Ferroptosis Phenotype Subgroups

To determine the DEGs between LGG samples of FRC1 and FRC2, we normalized the expression data of LGG samples by “voom” method of “limma” package [38] to transform RNA-Seq data ready for linear modelling and then calculated the statistical significance of DEGs using the “eBayes” function of “limma.” Genes with the adjusted *p* value less than 0.001 and |log2 (Fold Change)| > 1 were determined as significant DEGs.

### 2.7. Establishment of the FRscore

We constructed a ferroptosis-related scoring scheme for evaluating the ferroptosis trends or levels of individual LGG patients based on the method of principal component analysis (PCA). Primarily, we used the univariate Cox regression analysis to screen the prognostic DEGs between FRC1 and FRC2 LGG subgroups. Then, “Boruta” algorithm was used for the dimension reduction of the prognostic DEGs. Finally, principal components 1 (PC1) and 2 (PC2) of the PCA model of these prognostic DEGs were both extracted to establish the FRscore, which is similar to previous research, and equation was shown as the below:

(1) $$\begin{array}{c} \text{FRscore}=\Sigma \text{PC}1_{i}+\Sigma \text{PC}2i, \end{array}$$

where *i* is the expression of prognostic DEGs between FRC1 and 2.

### 2.8. Response of Immunotherapy Prediction: Tumor Immune Dysfunction and Exclusion (TIDE) and ESTIMATE

The Tumor Immune Dysfunction and Exclusion (TIDE) arithmetic was applied to evaluate the cancer immunologic escape mechanisms, including two basic factors of dysfunction of cytotoxic T lymphocyte (CTL) infiltration and exclusion of CTLs by immune suppressors [39]. The algorithm of ESTIMATE makes use of the special attributes of the transcriptional profiles to deduce the tumor cellularity as well as the tumor purity [40]. By using the ESTIMATE algorithm, we calculated the immune and stromal scores to predict the level of infiltrating immune and stromal cells, and these form the basis to infer tumor purity. Tumor tissues with abundant immune cell infiltration represented a higher immune score and lower level of tumor purity.

### 2.9. Acquisition of ICI Cohorts

To further validate the immunotherapy predictor role of FRscore, four clinical cohorts which contain cancer patients treated with ICI were used: IMvigor210 cohort (cancer patients treated with atezolizumab, anti-PDL1) [41], GSE91061 cohort (51 melanoma patients treated with nivolumab, anti-PD1) [42], PRJEB23709 cohorts (41 patients treated with pembrolizumab-nivolumab, anti-PD1 melanoma cohort; 32 patients treated with pembrolizumab-nivolumab combined with pembrolizumab, anti-PD1 and anti-CTLA4 melanoma cohorts) [43], and GSE100797 cohort (25 melanoma patients treated with anti-CTLA4) [43].

### 2.10. Prediction of TMZ Sensitivity

Temozolomide sensitivity data over 835 cancer cell lines (CCLs) and 482 CCLs were obtained from the Cancer Therapeutics Response Portal (CTRP version.2.0, https://portals.broadinstitute.org/ctrp) [44–46] and PRISM Repurposing dataset (19Q4, https://depmap.org/portal/prism/), respectively. The two databases offered the area under the curve (AUC) of dose–response scores, which negatively correlates with the drug sensitivity (higher AUC values represent lower sensitivity to TMZ). K nearest neighbor (k-NN) imputation was employed to fill the lost AUC values by using the R package “impute.” On account of the data of CCLs in both two databases were downloaded from the CCLE (Cancer Cell Line Encyclopedia) dataset; relative molecular CCLE data were utilized for subsequent TMZ-sensitivity analysis. Ridge regression, a model tuning method that is used to analyze any data that suffers from multicollinearity, was used to predict the AUC value of TMZ response for each LGG patient by applying the R package “pRRophetic” [47].

### 2.11. Cell Culture and Agents

SW1088 astrocytoma cell line was purchased from the American Type Culture Collection (ATCC) and cultured in the incubator with filtered air and 100% humidity, using culture medium which consisted of 89% Leibovitz's L-15 Medium (No. 30-2008; ATCC, USA), 10% fetal bovine serum (FBS) (Gibco, USA), and 1% penicillin and streptomycin (Gibco, USA). The erastin, a well-known ferroptosis inducer, was purchased from MedChemExpress company.

### 2.12. Reverse Transcription-Quantitative Polymerase Chain Reaction (RT-qPCR)

To detect the mRNA expression levels of the PIK3CA, AKT1, and MTOR of erastin-treated SW1088 cells, total RNA was extracted and used to synthesize cDNA by reverse-transcription reaction. Then, qPCR analysis was conducted to quantify the mRNA expressions. The procedure, instruments, and reagents of RT-qPCR analysis used in this research were in keeping with the previous research. Primers were obtained from a previous study [48] and listed as follows: PIK3CA forward: 5′-GGTTGTCTGTCAATCGGTGACTGT-3′, reverse: 5′-GAACTGCAGTGCACCTTTCAAGC-3′; AKT1 forward: 5′-TTCTGCAGCTATGCGCAATGTG-3′, reverse: 5′-TGGCCAGCATACCATAGTGAGGTT-3′; and MTOR forward: 5′-GCTTGATTTGGTTCCCAGGACAGT-3′, reverse: 5′-GTGCTGAGTTTGCTGTACCCATGT-3′.

### 2.13. Antibodies and Western Blot

The primary antibodies of PI3K (1 : 2000, 67071-1-Ig, Proteintech, China), AKT1 (1 : 1000, 10176-2-AP, Proteintech), pAKT1-S473 (1 : 2000, 66444-1-Ig, Proteintech), pAKT1-T308 (1 : 1000, #4056S, Cell Signaling Technology, USA), mTOR (1 : 5000, 66888-1-Ig, Proteintech), and GAPDH (1 : 5000, 10494-1-AP, Proteintech) and the secondary antibodies, including horseradish peroxidase- (HRP-) conjugated affinipure goat anti-rabbit IgG (1 : 2000, SA00001-2, Proteintech) and HRP-conjugated affinipure goat anti-mouse IgG (1 : 2000, SA00001-1, Proteintech), were used in western blot assay. The reagents and procedure of western blot assay in our research were in accord with our previous study.

## 3. Results

### 3.1. The Prognostic Role of FPI and the Genetic Alteration Landscape of Ferroptosis Regulators in LGGs

**Figure 1:**
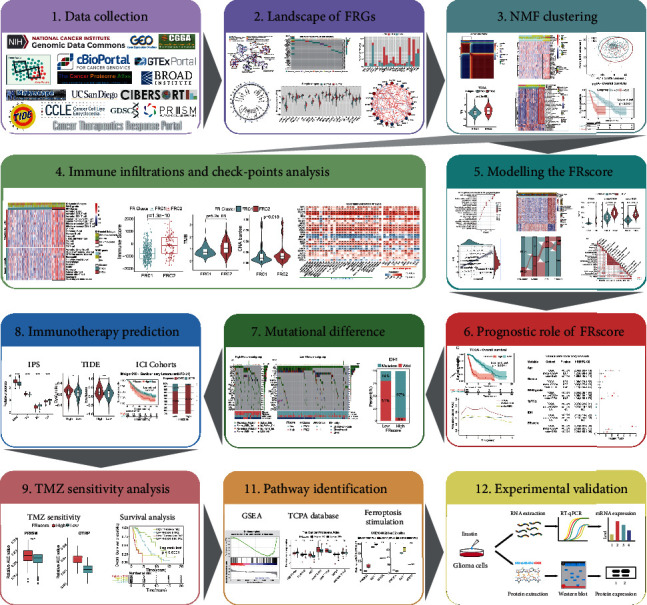
The design and workflow of this study.

**Figure 2:**
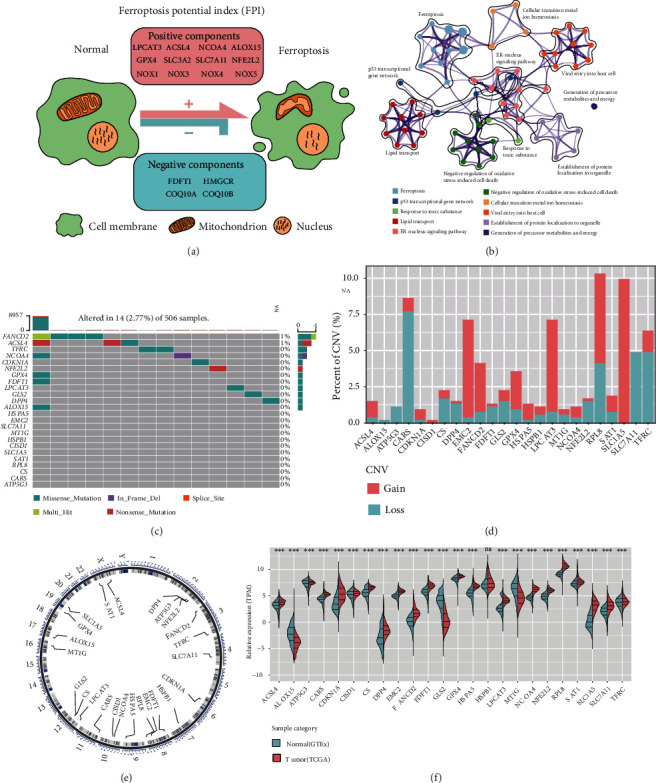
Genetic alteration landscape of ferroptosis regulators in lower-grade glioma. (a) The outline diagram of ferroptosis potential index (FPI) calculation regulated by positive components and negative components. (b) The Metascape pathway enrichment visualization of the 24 ferroptosis regulators showed the interactions among the enriched terms. (c) 14 of 506 LGG patients showed genomic mutations of 24 ferroptosis regulators, with a frequency of 2.77%, including missense mutations, in frame deletions, nonsense mutations, and multihits. (d) The bar plot represents the amplification (red) or deletion (blue) percent of the 24 FRGs in TCGA-LGG cohort. (e) The circle plot represents the locations of the 24 FRGs in the human chromosomes. (f) Comparison analysis showed the expression levels of 24 FRGs between human normal brain tissues from GTEx database and LGG samples from TCGA database. The labelled asterisk indicated the statistical *p* value (ns *p* > 0.05 and ^∗∗∗^*p* < 0.001).

To firstly uncover the ferroptosis regulators' functions in LGGs, it is necessary to investigate their genetic landscape in a multiomics aspect. The role of 24 FRGs, including the proferroptosis regulators, GLS2, FDFT1, EMC2, DPP4, CS, CARS, RPL8, ATP5G3, ALOX15, ACSL4, TFRC, SLC1A5, SAT1, NCOA4, and LPCAT3, and the antiferroptosis regulators, FANCD2, CISD1, CDKN1A, HSPB1, SLC7A11, NFE2L2, MT1G, HSPA5, and GPX4, and the ferroptosis potential index (FPI) were comprehensively investigated in LGGs. The workflow is summarized in Figure 1. Construction of FPI is mechanically shown in Figure 2(a) as previously described [10], with two core parts including positive components, LPCAT3, ACSL4, NCOA4, ALOX15, GPX4, SLC3A2, SLC7A11, NFE2L2, NOX1, NOX3, NOX4, and NOX5, and negative components, FDFT1, HMGCR, COQ10A, and COQ10B. Since the prognostic role of FPI in LGG is not known, survival analysis was performed in each LGG cohort, and the results indicated that FPI was a poor prognostic factor for LGG patients (Figure S1A-C). KEGG pathway enrichment analysis of the 24 FRGs was conducted using the Metascape webtool, and the significantly enriched pathways were visualized (Figure 2(b)) and concluded (Figure S1D). First, the frequency of somatic mutations in the 24 ferroptosis regulators was explored, and it was found that only 14 of 506 (2.77%) LGGs had FRG mutations (Figure 2(c)), suggesting that they remain at a low level in LGGs. Copy number variation (CNV) in the 24 ferroptosis regulators was also not prevalent in LGGs (Figure 2(d)). SLC1A5 had the most frequent CNV amplification (10%) in LGGs, and the most deleted ferroptosis regulator was CARS, which reached about 7.5%. Chromosomal localization of the 24 ferroptosis regulators was visualized (Figure 2(e)). To further analyze aberrant expression of the FRGs in LGGs, transcriptional data of normal brain tissues (NBTs) from the GTEx dataset were combined with LGG samples from the TCGA dataset. Results showed that while most FRGs were upregulated in LGG samples, GLS2 and MT1G were downregulated, and HSPB1 was similar in LGGs as normal brain tissues (Figure 2(f)).

Spearman correlation analysis indicated that the proferroptosis regulator, ACSL4, was positively correlated with the antiferroptosis regulators, GPX4 and HSPB1, and negatively correlated with the proferroptosis regulator, NCOA4. The proferroptosis regulator, RPL8, and antiferroptosis regulator, SLC7A11, were positively correlated in the three independent LGG cohorts (Figure S1E–G). These data show that the ferroptosis process within LGGs is carefully regulated. To further evaluate the prognostic role of FRGs, univariate Cox regression analysis was performed on the three LGG cohorts. By summarizing results from the three cohorts, CISD1 and FDFT1 were found to be likely protective regulators, and TFRC, FANCD2, LPCAT3, HSPA5, and HSPB1 were risk factors in LGGs (Figure S1H–J).

### 3.2. Recognition of Ferroptosis Regulator-Mediated Patterns in LGGs

**Figure 3:**
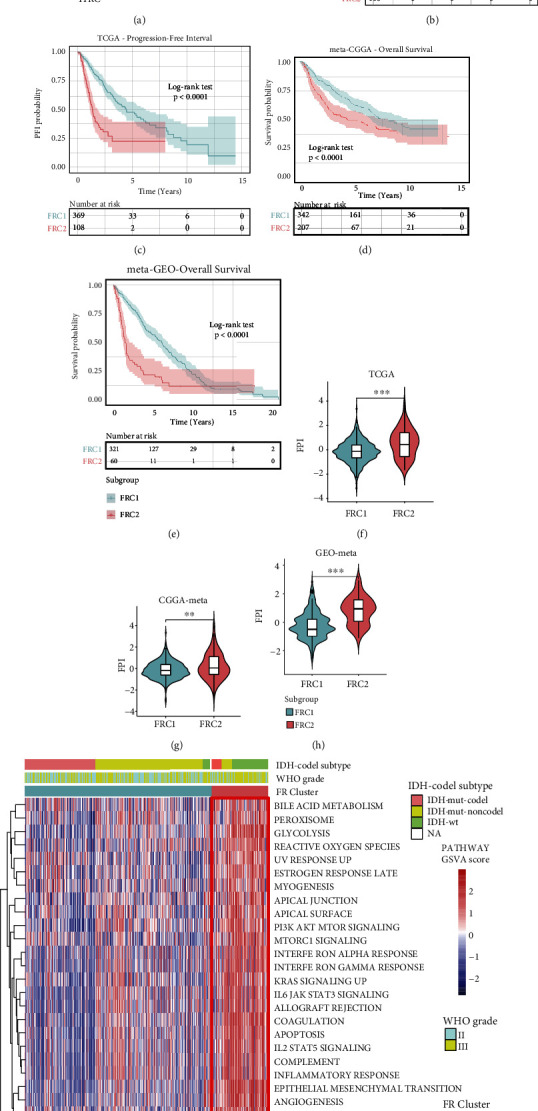
(a) The correlations between every two FRGs were shown. The FRGs (including promotive or antiferroptosis regulators) were described with circles with different colors. Proferroptosis regulator, blue; antiferroptosis regulator, red. The size of circle represents the statistical significance of prognostic effect of each FRG. The dot in the center of circle represents the prognostic role of FRGs, and the black dot represents risky factor while white dot represents protective factor. The lines between every two FRGs represent the significant Spearman correlations in different colors. Positive correlation, red; negative correlation, light blue. (b) Kaplan-Meier curves of overall survival (OS) for 477 LGG patients with two ferroptosis clusters in the TCGA-LGG cohort. The number of patients in FRC1 and FRC2 is 369 and 108, respectively (log-rank test, *p* < 0.0001). (c) Kaplan-Meier curves of progression-free interval (PFI) for 477 LGG patients with two ferroptosis clusters in the TCGA-LGG cohort (log-rank test, *p* < 0.0001). (d) Kaplan-Meier curves of overall survival (OS) for 549 LGG patients with two ferroptosis clusters in the meta-CGGA cohort. The number of patients in FRC1 and FRC2 is 342 and 207, respectively (log-rank test, *p* < 0.0001). (e) Kaplan-Meier curves of overall survival (OS) for 381 LGG patients with two ferroptosis clusters in meta-GEO cohort. The number of patients in FRC1 and FRC2 is 321 and 60, respectively (log-rank test, *p* < 0.0001). (f–h) The FPI was compared between FRC1 and FRC2 LGG subgroups in TCGA cohort (Wilcoxon rank-sum test, *p* < 0.0001), meta-CGGA cohort (Wilcoxon rank-sum test, *p* = 0.0015), and meta-GEO cohort (Wilcoxon rank-sum test, *p* < 0.0001). (i) The heat map shows the GSVA score of each hallmark in the LGG patients ordered by FRC cluster; the information of IDH-codel subtype and WHO grade was used for sample annotations. The labelled asterisk indicated the statistical *p* value (^∗∗^*p* < 0.01 and ^∗∗∗^*p* < 0.001).

To comprehensively illustrate how FRGs mediate ferroptosis-related function, a ferroptosis regulator gene network was designed using data from the TCGA cohort (Figure 3(a)). Results indicate that the ferroptosis was homeostatically regulated by FGGs in LGGs, and that cross-talk between pro- and antiferroptosis regulators is likely to play an important role in LGG tumorigenesis and progression. Based on these speculations, nonnegative matrix factorization (NMF), a group of consensus clustering algorithms, was performed to stratify LGG samples based on these 24 FRGs in order to find distinct ferroptosis regulator-mediated patterns. NMF clustering classified LGGs into two distinguishing clusters in the TCGA cohort, including 369 cases in ferroptosis regulator cluster 1 (FRC1) and 108 cases in ferroptosis regulator cluster 2 (FRC2) (Figure S2A, B). Clinical survival analysis showed that LGG patients in FRC2 exhibited better overall survival (OS, *p* < 0.0001, log-rank test) and a longer progression-free interval (PFI, *p* < 0.0001, log-rank test) than patients in FRC1 (Figures 3(b) and 3(c)). NMF clustering was also carried out in the meta-CGGA and meta-GEO cohorts, and the results of the survival analysis were found in the two meta-LGG cohorts (meta-CGGA: *p* < 0.0001, log-rank test; meta-GEO: *p* < 0.0001, log-rank test; Figures 3(d) and 3(e)). The ferroptosis potential index (FPI) was compared between the FRC1 and FRC2 LGGs in each cohort, and the FRC2 LGG group showed a significantly higher FPI (Figures 3(f)–3(h); TCGA: *p* < 0.0001; meta-CGGA: *p* = 0.0015; meta-GEO: *p* < 0.0001, Wilcoxon test).

Principal component analysis (PCA) used to verify differences between FRC1 and FRC2 based on whole transcriptome data could not differentiate between the subgroups (Figure S2F–H). These findings indicated that the FRC cluster method provides a novel way to classify LGGs without a significant difference in transcriptional characterization.

### 3.3. Ferroptosis Patterns Are Characterized by Distinct Immune Landscapes

**Figure 4:**
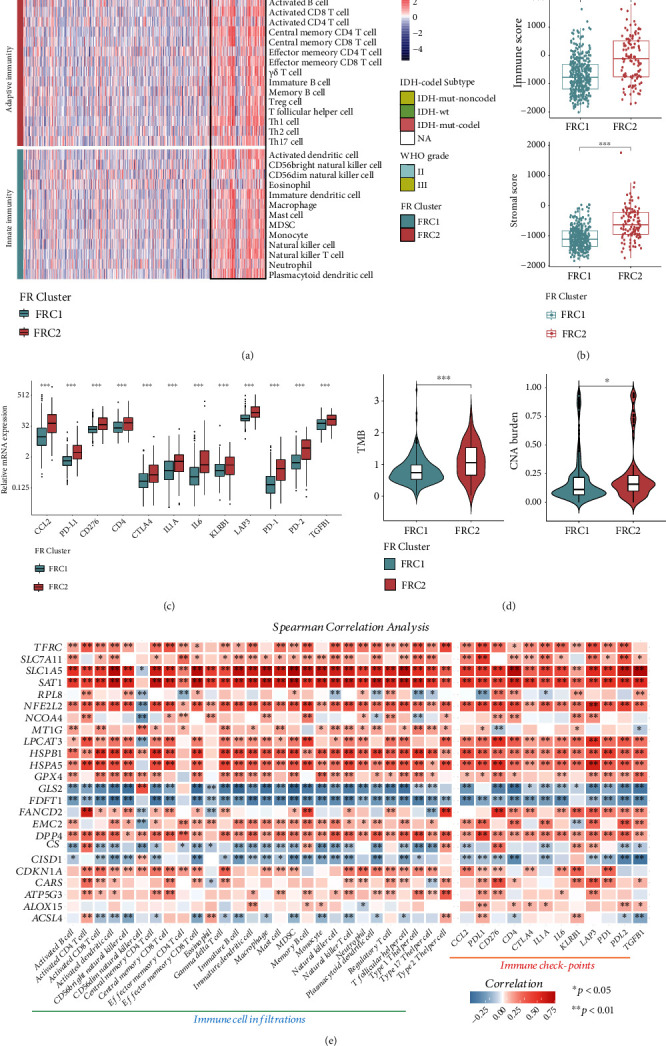
(a) The heat map represents the 22 types of immune cell infiltration levels calculated by ssGSEA algorithm ordered by FRC cluster; the information of IDH-codel subtype and WHO grade was used for sample annotations. (b) Immune score and stromal score overall levels between FRC1 and FRC2 were analyzed and visualized. (c) The expression levels of twelve immune checkpoint genes were compared between FRC1 and FRC2 clusters. (d) The tumor mutation burden (TMB) and copy number variation (CNA) burden were analyzed between FRC1 and FRC2 clusters. (e) The Spearman correlation heat map shows the correlations between the 24 FRG expressions and the 22 types of immune cell infiltrations or the 12 immune checkpoint gene expressions. Red represents positive correlation, and blue represents negative correlation.

To further characterize the underlying molecular mechanisms of ferroptosis-related clusters, gene set enrichment analysis (GSVA) was implemented to score hallmark gene sets in each LGG sample. GSVA revealed that immune activation-associated hallmarks such as the IFN-gamma/alpha response; allograft rejection and inflammatory response; classical carcinogenic activation pathways including the PI3K-AKT-mTOR, KRAS, and TNF signaling pathways; and cancer malignancy phenotypes like glycolysis, epithelial-mesenchymal transition (EMT), angiogenesis, and hypoxia were significantly enriched in the LGG FR2 subgroup (Figure 3(i)). To further investigate distinctions between the innate tumor microenvironments of FRC1 and 2, 28 immune cell infiltration levels were quantified and compared between two ferroptosis regulator clusters using the ssGSEA algorithm (Figure 4(a)). FRC2, the LGG subgroup with a poor prognosis, showed higher immune cell infiltration than the FRC1 LGG subgroup (Figure S3A). In addition, the FRC2 subgroup had higher immune and stromal scores (Figure 4(b), immune score: *p* < 0.001; stromal score: *p* < 0.001, Wilcoxon test) which is indicative of higher immune and stromal cell infiltrations in FRC2 and consistent with the ssGSEA results. Thus, the FRC2 LGG subgroup was defined as “higher-immunity” or “hot” while the FRC1 LGG subgroup was defined as “lower-immunity” or “cold.” CIBERSORT immune cell infiltration analysis also showed that FRC2 was significantly more enriched with immune cells and plasma cells, CD8+ T cells, T helper cells, M0 and M1 macrophages, and activated dendritic cells (Figure S3B). The levels of 12 immune checkpoint genes, tumor mutation burden (TMB), copy number alteration (CNA) burden, and altered LOH fraction were also higher in the FRC2 subgroup (Figures 4(c) and 4(d) and Figure S3C). However, the differential amount of neoantigen between the two FRC clusters was not statistically significant (Figure S3D), and the stemness index of the FRC1 cluster was significantly higher than it was for FRC2 (Figure S3E). These results indicate that LGGs in the FRC2 cluster are characterized by high immune infiltration and ferroptosis, high TMB, CNA burden, altered LOH fraction and immune check-point gene expression, and low stemness index.

To assess the association between the 24 FRGs and immune cell infiltration or immune check-point genes (ICPGs), a Spearman correlation analysis was performed between each FRG and the proportion of immune cells and ICPG. SLC1A5 and SAT1 correlated strongly with immune cell infiltration and ICPGs, while GLS2 and FDFT1 were negatively associated with both (Figure 4(e)). A previous study showed that SLC1A5, also named ASCT2, which encodes the glutamine transporter, activates naive T cell activation, and SLC1A5 deficiency can inhibit Th1 and Th17 cell induction and reduce inflammatory T cell responses *in vivo* [49]. These data indicate that SLC1A5 is vital to T cell activation and responses and supports a strong positive correlation between SLC1A5 expression and Th1/17 cell infiltration.

### 3.4. Identifying DEGs between Ferroptosis Patterns in LGGs

**Figure 5:**
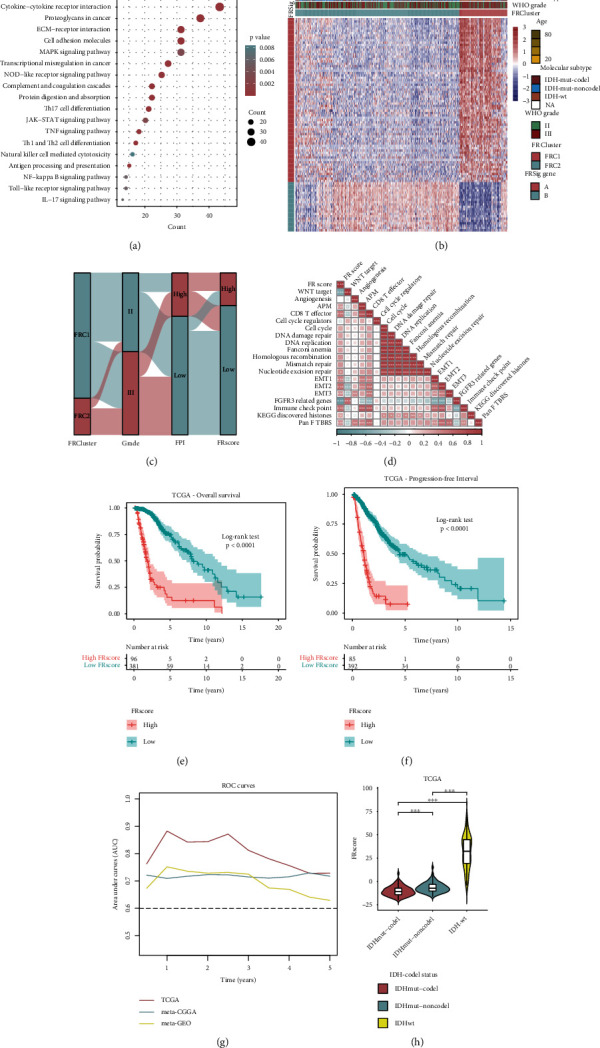
(a) KEGG pathway enriched terms of FRC2 upregulation genes. (b) The heat map showed the expressions of prognostic FRSig genes, which were screened by univariate Cox regression analysis, between two FRC clusters. The LGG samples were annotated with the information of age, molecular subtype, and WHO grade. (c) Alluvial diagram of FRC clusters in group with distinct WHO grade, FPI level, and FRscore. (d) Spearman correlation analysis between FRscore and well-established biological signatures. (e, f) Kaplan-Meier curves for low- and high-FRscore LGG subgroups in the TCGA cohort showed LGG patients with higher FRscores have shorter OS (e) and PFI (f) time. (g) A line chart showed the area under curve (AUC) values of FRscore in the three LGG cohorts for predicting the 1–5-year OS. (h) Distribution of FRscore in distinct IDH-codeletion subtypes in the TCGA cohort, and the FRscore levels between every two molecular subgroups were compared by Wilcoxon rank-sum test. The labelled asterisk indicated the statistical *p* value (ns *p* > 0.05, ^∗^*p* < 0.05, ^∗∗^*p* < 0.01, and ^∗∗∗^*p* < 0.001).

FRG-based NMF clustering classified LGG patients into two ferroptosis and immune phenotypes, but the underlying fluctuation in expression and genetic malformation between the two FRC clusters remain unknown. To further investigate the role of ferroptosis in LGGs, potential ferroptosis-associated transcriptional alterations between the two FRC clusters were assessed. Of the 2,508 DEGs screened, 2,109 DEGs were upregulated in FRC2 LGGs, and 399 DEGs were upregulated in FRC1. Enrichment analysis of the KEGG pathway showed that multiple oncogenic pathways including PI3K-Akt, MAPK, JAK-STAT, TNF, and NF-kappa B signaling were enriched in the FRC2 cluster LGGs (Figure 5(a)), correlating with GSVA results.

### 3.5. Establishment and Clinical Relevance of the FRscore

Previous findings have demonstrated the relevance of immune infiltration during ferroptosis in LGG, but stratification of LGG patients was based on a population cohort and lacked individual patient-level detail. Hence, a marking scheme was developed named FRscore, based on FRSig genes, to measure ferroptosis trends in individual LGG patients. The expression matrices of the 2,508 DEGs were extracted, and the univariate Cox regression and Boruta algorithm were combined to develop the FRscore. Ninety-one FRSig genes were identified and used to construct an FRscore for each LGG patient. The correlation between ferroptosis levels and expression of the 91 FRSig genes is shown in the heat map (Figure 5(b)). To further characterize the association among the FRCluster, WHO grade, FPI, and FRscore, an alluvial diagram was designed (Figure 5(c)). Spearman correlations between well-known biological signatures and the FRscore were assessed, and the results indicated that the FRscore correlated strongly with immune activation, including antigen process machinery (APM), CD8 T effector and immune checkpoint, and EMT-related (EMT1/2/3) signatures, but was negatively associated with stromal-relevant signatures (FGFR3-related genes and WNT target). Furthermore, survival results showed that LGG patients with higher FRscores survived for less time in all three LGG cohorts (Figures 5(e) and 5(f) and Figure S4A, B; TCGA-overall survival: *p* < 0.0001; TCGA-progression-free interval: *p* < 0.0001; meta-CGGA-overall survival: *p* < 0.0001; meta-GEO-overall survival: *p* < 0.0001; log-rank test), and ROC curves showed that the FRscore possessed strong prognostic ability (Figure 5(g)). These data indicate that the FRscore may be a promising prognostic biomarker for LGG. In addition, IDH-wild LGG patients exhibited the highest FRscore, LGG patients with IDH-mutation and 1p/19q-codeletion status showed the lowest FRscore (Figure 5(h) and Figure S4C), and higher FRscores were observed in the FRC2 LGG patients than the FRC1 subgroup (Figure S4D). In addition, LGG patients with higher FRscores had a higher FPI index, and the FRscore showed a strong Pearson correlation with the FPI index in LGGs (Figure S4D–F). High-FRscore LGGs were also correlated with higher TMB, higher LOH fraction, higher CNA burden, and a lower stemness index (Figure S4I–L), and the neoantigen number was not differentially distributed between low- and high-FRscore LGG patients (Figure S4M).

**Figure 6:**
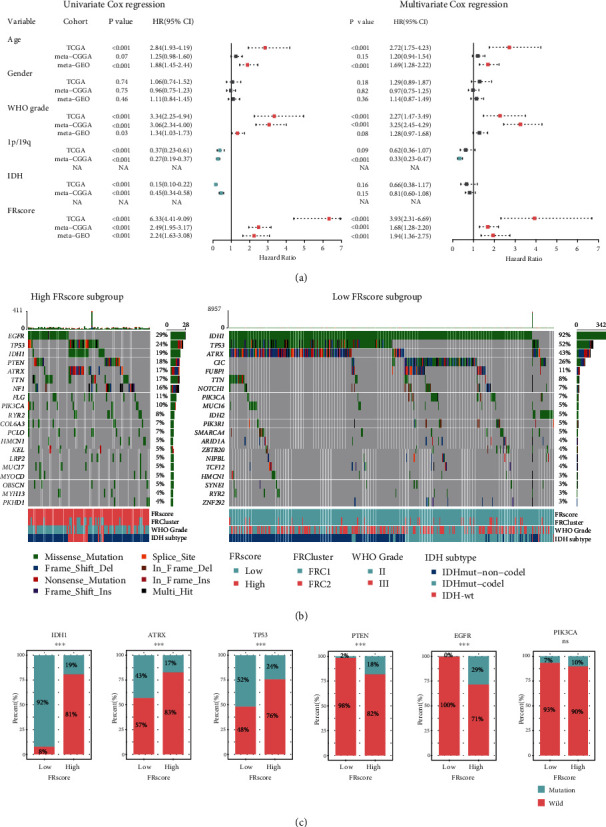
(a) Univariate and multivariate Cox regression indicated that the FRscore was an independent prognostic predictor for LGG patients. (b) Mutational landscape of top 20 somatic mutation genes in TCGA-LGG stratified by low- and high-FRscore. LGG patients were annotated by FRscore subgroup, FRC cluster, WHO grade, and IDH-codeletion subtype. (c) Fractions of patients with gene mutation in low- and high-FRscore LGG subgroups. Genes of IDH, ATRX, TP53, PTEN, EGFR, and PIK3CA were analyzed. The labelled asterisk indicated the statistical *p* value (^∗∗∗^*p* < 0.001). The labelled asterisk indicated the statistical *p* value (ns *p* > 0.05 and ^∗∗∗^*p* < 0.001).

These results indicate that the FRscore was significantly associated with cancer immune activation and the clinical outcomes of LGG patients. Thus, the independent prognostic role of the FRscore in the TCGA-training and two metavalidation cohorts were also assessed. LGG patients were grouped into high- and low-FRscore subgroups using the best cutoff value in the survival analysis. Univariate and multivariate Cox regression analyses were used to evaluate the prognostic ability of the FRscore, and the results showed that the FRscore was a stronger and more independent risk indicator of LGGs than other clinicopathological features, including age of diagnosis, gender, WHO grade, IDH status, and 1p/19q codeletion status (Figure 6(a)).

### 3.6. Mutational Landscapes of LGG Patients with Distinct FRscore Levels

To better understand the association between genomic alterations and the FRscore, two waterfall plots were designed to show the gene mutation landscape of the two LGG subgroups (low-FRscore vs. high-FRscore) (Figure 6(b)). Results indicated that the low-FRscore LGGs had higher IDH1 (92% vs. 19%), ATRX (43% vs. 175), and TP53 (52% vs. 24%) mutational proportions and lower PTEN (2% vs. 18%) and EGFR (0% vs. 29%) mutations than the high-FRscore LGGs (Figure 6(c)). These data showed that several frequent mutational LGG genes were differentially distributed between the low- and high-FRscore LGG groups and indicated that some mutations might be associated with sensitivity or resistance of LGGs to ferroptosis.

### 3.7. Role of the FRscore in Predicting Response to Immunotherapy

**Figure 7:**
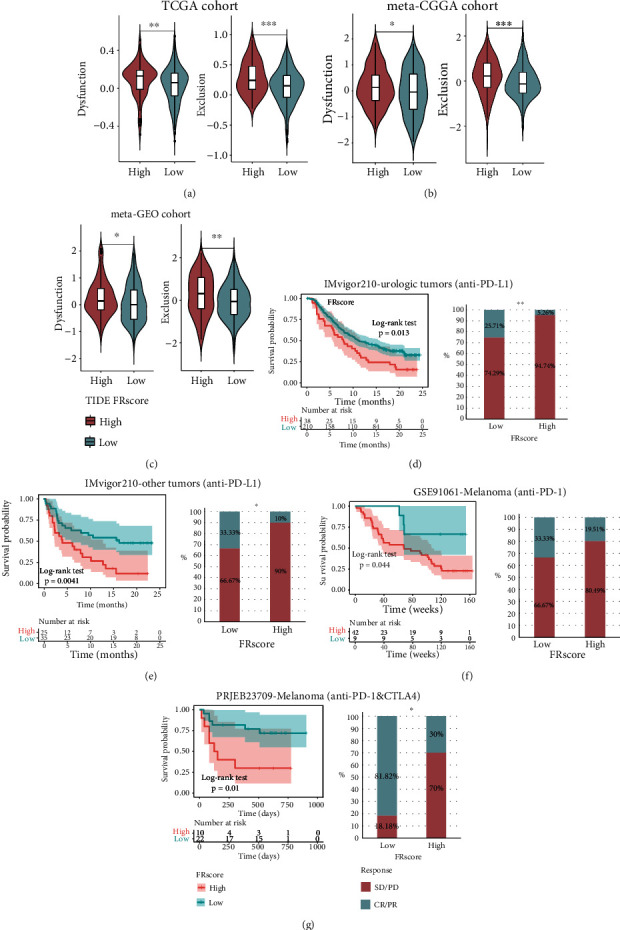
(a–c) The distributions of dysfunction and exclusion scores were compared between low- and high-FRscore LGG subgroups in the three LGG cohorts (Wilcoxon rank-sum test). (d) Kaplan-Meier curves for low- and high-FRscore patient groups in urologic tumors (including bladder, kidney, and ureter tumors) of IMvigor210 cohort (anti-PD-L1) and the fraction of urologic tumor patients with response to anti-PD-L1 therapy in low- and high-FRscore subgroups of IMvigor210 cohort. (e) Kaplan-Meier curves for low- and high-FRscore patient groups in other tumors (including liver, lung, and lymph node tumor) of IMvigor210 cohort (anti-PD-L1) and the fraction of other tumor patients with response to anti-PD-L1 therapy in low- and high-FRscore subgroups of IMvigor210 cohort. (f) Kaplan-Meier curves for low- and high-FRscore patient groups in GSE91061 cohort (anti-PD-L1, melanomas) and the fraction of melanoma patients with response to anti-PD-1 therapy in low- and high-FRscore subgroups of GSE191061 cohort. (g) Kaplan-Meier curves for low- and high-FRscore patient groups in PRJEB23709 cohort (anti-PD-L1, melanomas) and the fraction of melanoma patients with response to anti-PD-1 therapy in low- and high-FRscore subgroups of PRJEB23709 cohort. The labelled asterisk indicated the statistical *p* value (^∗^*p* < 0.05, ^∗∗^*p* < 0.01, and ^∗∗∗^*p* < 0.001).

Given that the FRscore was significantly associated with immune activation signatures, its potential role in cancer immunotherapy was also assessed. Immune checkpoint inhibitors, like anti-PD-L1/PD-1/CTLA-4, have great therapeutic potential in antitumor therapy. As compared to previously identified TMB, MSI, and PD-L1, artificial predictors like TIDE are extensively used to assess responses to immunotherapy. Analyses showed that the FRscore was tightly correlated with the dysfunction and exclusion scoring of the TIDE algorithm in the three LGG cohorts (Figures 7(a)–7(c)). A high-FRscore was associated with higher dysfunction and exclusion scoring, suggesting that high-FRscore LGGs may benefit less from immunotherapy than low-FRscore LGGs.

Immunotherapy cohort validation was then conducted by combining clinical information and transcriptional data to evaluate the FRscore ability to predict responsiveness to immunotherapy. The FRscore was calculated for each patient in the four ICI cohorts based on their transcriptional profile prior to ICI treatment. Since the IMvigor210 cohort contains several types of cancer patients, they were divided into the genitourinary tumor subgroup (*n* = 248) and other tumor subgroup (*n* = 60). Kaplan-Meier survival analysis showed that the FRscore stratified urologic tumor patients (Imvigor210 cohort) into two subgroups with distinct clinical outcomes, with the high-FRscore subgroup showing a poor response to anti-PD-L1 therapy (Figure 7(d), *p* = 0.013, log-rank test). Similar results were also found in the other tumor cohort (Figure 7(e),*p* = 0.0041, log-rank test) and two melanoma cohorts (Figures 7(f) and 7(g); GSE91061, anti-PD-1 cohort:*p* = 0.044; and PRJEB23709, anti-PD-1 and anti-CTLA4 mixed cohorts:*p* = 0.01; log-rank test). These findings showed that the FRscore correlated strongly with responsiveness to immunotherapy and may be used for the clinical prognosis of cancer patients.

### 3.8. TMZ Sensitivity Analysis of the FRscore

**Figure 8:**
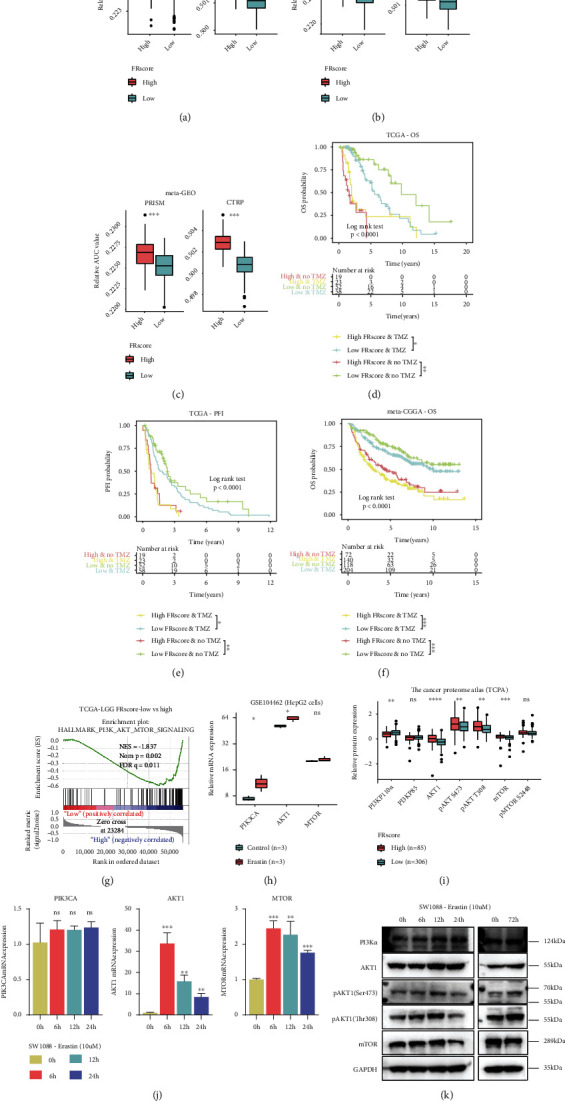
(a–c) The boxplots compared the AUC values of TMZ calculated by ridge regression using the two databases of PRISM and CTRP between low- and high-FRscore in the TCGA (a), meta-CGGA (b), and meta-GEO (c) LGG cohorts (Wilcoxon rank-sum test). (d–f) Kaplan-Meier curves represent the LGG subgroups of patients with different FRscore levels and TMZ therapy status to reflect the OS (d) and PFI (e) statuses in the TCGA cohort and OS status in the meta-CGGA cohort (f). (g) GSEA result showed that the hallmark of PI3K-AKT-mTOR pathway was enriched in the high-FRscore LGG subgroup. (h) The PIK3CA, AKT1, and MTOR mRNA expressions were compared between HepG2 cells treated with erastin and control cells using data from GSE104462 (Students' *t*-test). (i) The PI3K-AKT-mTOR pathway-associated protein expressions of matched LGG samples from different FRscore subgroups were compared and visualized by boxplots (Wilcoxon rank-sum test). (j) The bar plots represent the mRNA expression levels of PIK3CA, AKT1, and MTOR in SW1088 cells treated with 10 *μ*M erastin for 0, 6, 12, and 24 hours. (k) The bands showed the PI3K-AKT-mTOR pathway-associated protein expression levels in SW1088 cells treated with 10 *μ*M erastin for 0, 6, 12, 24, and 72 hours. The labelled asterisk indicated the statistical *p* value (ns *p* > 0.05, ^∗^*p* < 0.05, ^∗∗^*p* < 0.01, and ^∗∗∗^*p* < 0.001).

Given that LGG immunotherapy is not ecumenical and TMZ is the mainstream chemotherapeutic drug for LGG, the association between TMZ sensitivity and ferroptosis patterns was assessed in LGG patients. The AUC value of each LGG patient was calculated using ridge regression based on TMZ sensitivity data from the Cancer Therapeutics Response Portal (CTRP) and the PRISM Repurposing dataset. The ferroptosis pattern was significantly associated with TMZ sensitivity in LGG patients. High-FRscore LGG patients had higher AUC values than low-FRscore LGG patients in all LGG cohorts (Figures 8(a)–8(c)), strongly suggesting that LGG patients with higher FRscores may be more resistant to TMZ therapy. Along with the TMZ therapy information in the TCGA and meta-CGGA cohorts, the prognostic ability of FRscore in TMZ-treated and untreated LGG subgroups was analyzed. Survival analysis indicated that the FRscore had a prognostic role in both TMZ-treated and TMZ-untreated LGG subgroups (Figures 8(d)–8(f)), showing that it can be a robust and accurate prognostic biomarker for both TMZ-treated and untreated LGG patients.

### 3.9. Ferroptosis Alters the PI3K-AKT-mTOR Signaling Pathway

To better understand the underlying molecular mechanisms that associate ferroptosis with clinical outcomes, changes in ferroptosis-induced signaling pathways were assessed. Yi et al. [50] showed that oncogenic promotion of the PI3K-AKT-mTOR pathway reduced ferroptosis using SREBP-mediated lipogenesis. Similar to the association between the GSVA and KEGG pathways and the FRC2 LGG subgroup, the PI3K-AKT-mTOR pathway correlated significantly with the FRscore using gene set enrichment analysis (Figure 8(g)). The public transcriptional data of erastin-treated HepG2 cells was also assessed, and PIK3CA and AKT1 mRNA expressions increased significantly when treated with the ferroptosis inducer, Ersatin (Figure 8(h)). To measure PI3K-AKT-mTOR protein pathway expression associated with distinct ferroptosis levels, TCPA level-3 protein expression was compared to PI3K-AKT-mTOR pathway protein expression in the low- and high-FRscore LGG subgroups. TCPA data indicated that AKT1, pAKT-S473, pAKT-T308, and mTOR protein expression increased significantly in high-FRscore LGGs but PI3K-P100a decreased, and PI3K-P85 and pMTOR-S2488 were not statistically different (Figure 8(i)).

Taken together, these data suggest that the intraglioma ferroptosis level may affect cellular survival, proliferation, and drug sensitivity by regulating the PI3K-AKT-mTOR pathway. The SW1088 cell line was treated with 10 *μ*M erastin, and PIK3CA-AKT-mTOR mRNA and protein expressions were measured by RT-qPCR and western blot. RT-qPCR data showed increases in AKT1 and MTOR mRNA expression. A 6-hour induction with erastin led to a 33-fold increase in AKT1 mRNA expression; however, PIK3CA mRNA expression was unaltered by stimulation of ferroptosis (Figure 8(j)). To prepare for western blot, SW1088 cells were cultured and treated with 10 *μ*M erastin for 0, 6, 12, and 24 hours. Results showed that PI3Ka, pAKT1-S473, and pAKT1-T308 protein expressions increased within 24 hours of ferroptosis induction, but mTOR protein expression was downregulated during the same time period. To assess the effect of prolonged ferroptosis stimulation on LGG cells, SW1088 cells were treated with 10 *μ*M erastin for 72 hours (cell culture medium containing 10 *μ*M erastin was replaced after each 24-hour period). Findings showed that pAKT1-S473 and pAKT1-T308 protein expressions were still upregulated (Figure 8(k)).

## 4. Discussion

Since ferroptosis was first defined, its function in cancer progression, chemotherapy, and immunotherapy has attracted great attention [51]. Increasing evidence indicates that iron-dependent programming death plays a vital role in shaping the cancer microenvironment and regulating antitumor immunity [52–55]. Many studies have uncovered complex roles for ferroptosis regulators in modulating the tumor microenvironment, but their role in regulating ferroptosis has not been comprehensively analyzed in LGG [10, 56]. A thorough study focused on recognizing distinct ferroptosis patterns in the TME can provide clues about how ferroptosis regulates antitumor immunity and inform more creative immunotherapy strategies.

Two LGG clusters characterized by entirely distinct ferroptosis levels, immune cell infiltration, and clinical prognosis were defined in this study. FRC1 was characterized by an immune-excluded or desert phenotype, lower ferroptosis levels, and a favorable clinical prognosis, while FRC2 LGGs had higher immune cell infiltration with an immune-inflamed phenotype, higher ferroptosis levels, and a poorer prognosis. Higher immune and stromal scores also indicated greater immune infiltration in FRC2 LGGs, along with higher immune checkpoint gene expression and TMB, suggesting that classification of ferroptosis is associated with responsiveness to immunotherapy. KEGG enrichment analysis was conducted to define the underlying mechanisms that might explain the differences between the two clusters. Several oncogenic pathways, including PI3K-AKT-mTOR, MAPK, and JAK-STAT, along with immune processes like Th1, Th2, and Th17 cell differentiation and natural killer cell-mediated cytotoxicity, were enriched in FRC2 LGGs. A previous study [50] showed that activation of PI3K-AKT-mTOR suppressed ferroptosis in cancer cells; however, this study showed a strong correlation between ferroptosis and activation of the PI3K-AKT-mTOR pathway. Thus, it was hypothesized that ferroptosis could activate PI3K-AKT-mTOR signaling pathway to suppress ferroptosis induction. Study findings supported this hypothesis and showed that SLC1A5, a proferroptosis regulator, is required for induction of Th1 and Th17 cells, reinforcing the use of KEGG enrichment analysis warrants further study.

The FRscore was developed as an individual scoring scheme for LGG patients based on 91 identified FRSig genes and was found to correlate with the status of cancer immunity as well as LGG prognosis. The ferroptosis potential index (FPI), a previously identified factor representing the level of ferroptosis, was also strongly associated with the FRscore; this indicated that the FRscore could also reflect the ferroptosis level of LGGs. Further analysis showed that the FRscore correlated with response to immunotherapy and TMZ sensitivity, indicating that it could be applied to a wider range of clinical outcomes among LGG patients. Finally, the PI3K-AKT-mTOR pathway was shown to be involved in ferroptosis induction in LGG cells, showing that glioma cells could respond to ferroptosis by inducing intrinsic oncogenic activity.

Different effects caused by distinct ferroptosis levels may explain its dual role during cancer. When induction is not enough to cause programmed death, malignant cells can respond to stimulation by activating intrinsic oncogenic pathways that withstand ferroptosis pressure; however, sufficiently inducing ferroptosis to cause cell death may be a promising option for cancer therapy. Immunotherapy has made progress in clinical trials of glioma patients but developing methods for managing these patients requires additional research. The ferroptosis-related scoring system developed here is a promising indicator for LGG immunotherapy. In addition, the use of FRscore to assess responsiveness to TMZ and other treatments has promise for LGG patients. This study showed that a higher FRscore not only indicated resistance to TMZ therapy for LGG patients but also represented a poor prognosis in LGG patients either treated or not treated with TMZ. An increase in erastin-induced ferroptosis was shown to enhance the migration of GBM cells, and TMZ-resistant GBM cells were more sensitive to erastin induction [6]. However, the correlation between ferroptosis and TMZ resistance is not fully understood, and additional research may provide some clues about the underlying mechanism.

This study comprehensively assessed ferroptosis patterns among 1,407 LGG patients and showed that the high level of ferroptosis seen in FRC2 LGG patients was the result of elevated immune cell infiltration that indicated a poor prognosis. The FRscore also showed extensive clinical promise for LGG patients during TMZ chemotherapy or immunotherapy management. This comprehensive integrated study indicated that intraglioma ferroptosis levels may predict formation of the immune microenvironment and regulation of cancer immunity. Calculating the FRscore for individual patients based on their pathological specimens may help to predict disease outcomes and responsiveness to treatment.

## 5. Conclusion

Our research highlights the critical role of ferroptosis in TME formation and shaping, and quantitatively assessing ferroptosis levels in individual tumors can help to define the intratumor microenvironment and formulate precise treatment strategies for LGG patients.

## Figures and Tables

**Table 1:** Summary of clinical characteristics of patients with colon cancer in four datasets.

Characteristic	TCGA dataset	CGGA_325 dataset	CGGA_693 dataset	GSE16011 dataset	GSE61374 dataset	Rembrandt dataset
No. of patients	477	170	379	103	137	141
Platform	Illumina RNAseq	Illumina HiSeq	Illumina HiSeq	Affymetrix U133 plus 2.0 array	Affymetrix U133 plus 2.0 array	Affymetrix U133 plus 2.0 array
Age (years)						
Range	14-87	10-74	11-69	23-81	21-80	17-87
Median	41	39	40	44	41	42
Gender						
Female	216	65	167	36	53	47
Male	261	105	212	67	84	72
Unknown	0	0	0	0	0	22
WHO grade						
II	231	97	153	22	61	76
III	246	73	226	81	76	65
IDH mutation status						
Yes	389	125	262	45	115	
No	85	44	80	37	22	
Unknown	3	1	37	21	0	
1p/19q codeletion status						
Yes	156	55	122	37	37	
No	321	113	255	39	100	
Unknown	0	2	2	27	0	
Overall survival (year)						
Range	0.10-17.60	0.18-13.18	0.14-13.78	0.19-20.68	0-17.7	0.08-20.69
Median	1.98	6.05	3.98	3.3	4.4	3.16
Progression-free interval (year)						
Range	0.02-14.39					
Median	1.54					

## Data Availability

Data associated with this study are summarized in the manuscript or included in supplemental information. All data used in this work can be acquired from the UCSC Xena website (https://xenabrowser.net/datapages/), Chinese Glioma Genome Atlas (CGGA, http://www.cgga.org.cn/), and the website of Gene Expression Omnibus (GEO, https://www.ncbi.nlm.nih.gov/gds).

## Abbreviations

LGG: Lower-grade glioma
GBM: Glioblastoma
CNS: Central nervous system
GEO: Gene Expression Omnibus
TCGA: The Cancer Genomic Atlas
CGGA: Chinese Glioma Genomic Atlas
ssGSEA: Single sample gene set enrichment analysis
TMZ: Temozolomide
NMF: Nonnegative matrix factorization
GSVA: Gene Set Variation Analysis
PCA: Principal component analysis
ICI: Immune check-point inhibitors
CTL: Cytotoxic T lymphocyte
TME: Tumor microenvironment
RMA: Robust multiarray averaging
TMB: Tumor mutation burden
TIDE: Tumor Immune Dysfunction and Exclusion
CNV: Copy number variation
DEGs: Differential expression genes
ROC: Receiver operator characteristic
CIBERSORT: Cell-type Identification By Estimating Relative Subsets Of RNA Transcripts.

## Supplementary Materials

Supplementary Materials Figure S1: (A–C) Kaplan-Meier curves revealed the FPI could stratified LGG patients into low- and high-FPI subgroup with distinct prognosis in the TCGA (A, log-rank test, *p* < 0.0001), meta-CGGA (B, log-rank test, *p* < 0.0001), and meta-GEO (C, log-rank test, *p* < 0.048) cohorts. (D) The significant enriched terms of the 24 FRGs in Metascape enrichment analysis. (E–G) The Spearman correlation heat maps showed the correlations between every two FRGs in the TCGA (E), meta-CGGA (F), and meta-GEO (G) cohorts. (H–J) The forest plots showed the prognostic role of the 24 FRGs in the TCGA (H), meta-CGGA (I), and meta-GEO (J) cohorts. The red name represents antiferroptosis regulator, and blue means proferroptosis regulator. Figure S2: (A) The correlation between cophenetic, dispersion, evar, residuals, rss, silhouette, and sparseness coefficients with the number of unsupervised NMF clusters. (B) Heat map of two unsupervised NMF clusters for ferroptosis regulators in the TCGA-LGG cohort. (C–E) Heat maps depict the expression levels of 24 ferroptosis regulator genes in TCGA (C), meta-CGGA (D), and meta-GEO (E) cohorts and annotated with FRCluster, age, gender, and grade information. (F–H) Principal component analysis (PCA) of the FRC clusters based on the whole transcriptional profile in the TCGA (F), meta-CGGA (G), and meta-GEO (H) cohorts. Figure S3: (A) The comparisons of 28 immune cell fractions quantified by ssGSEA algorithm between FRC1 and FRC2 LGG subgroups (Wilcoxon rank-sum test). (B) The comparisons of CIBERSORT immune cell fractions between FRC1 and FRC2 LGG subgroups (Wilcoxon rank-sum test). (C–E) Neoantigen (C), LOH fractions (D), and stemness index (E) were compared between FRC1 and FRC2 LGG subgroups (Wilcoxon rank-sum test). The labelled asterisk indicated the statistical *p* value (ns *p* > 0.05, ^∗^*p* < 0.05, ^∗∗^*p* < 0.01, and ^∗∗∗^*p* < 0.001). Figure S4: (A, B) Kaplan-Meier curves for low- and high-FRscore LGG subgroups in the meta-CGGA cohort (A) and meta-GEO cohort (B) present LGG patients with higher FRscores have poorer prognosis. (C) Distribution of FRscore in distinct IDH-codeletion subtypes in the meta-CGGA cohort, and the FRscore levels between every two molecular subgroups were compared by Wilcoxon rank-sum test. (D) Comparison of FRscores between FRC1 and FRC2 LGG subgroups in the three LGG cohorts (Wilcoxon rank-sum test). (E) Comparison of FPI levels between FRC1 and FRC2 LGG subgroups in the three LGG cohorts (Wilcoxon rank-sum test). (F–H) Spearman correlation analysis showed the positive correlation between FPI and FRscore in the three LGG cohort. (I–M) Tumor mutation burden (TMB, I), loss of heterozygosity (LOH) fraction (J), copy number alteration (CNA) burden (K), stemness index (L), and neoantigen (M) levels were compared between low- and high-FRscore LGG subgroups (Wilcoxon rank-sum test). The labelled asterisk indicated the statistical *p* value (ns *p* > 0.05 and ^∗∗∗^*p* < 0.001).

## References

[1] Yang W. S., Stockwell B. R. (2016). Ferroptosis: death by lipid peroxidation. *Trends in Cell Biology*.

[2] Stockwell B. R., Friedmann Angeli J. P., Bayir H. (2017). Ferroptosis: a regulated cell death nexus linking metabolism, redox biology, and disease. *Cell*.

[3] Yang W. S., SriRamaratnam R., Welsch M. E. (2014). Regulation of ferroptotic cancer cell death by GPX4. *Cell*.

[4] Yu H., Guo P., Xie X., Wang Y., Chen G. (2017). Ferroptosis, a new form of cell death, and its relationships with tumourous diseases. *Journal of Cellular and Molecular Medicine*.

[5] Xie Y., Zhu S., Song X. (2017). The tumor suppressor p53 limits ferroptosis by blocking DPP4 activity. *Cell Reports*.

[6] Liu H. J., Hu H. M., Li G. Z. (2020). Ferroptosis-related gene signature predicts glioma cell death and glioma patient progression. *Frontiers in Cell and Development Biology*.

[7] Louandre C., Ezzoukhry Z., Godin C. (2013). Iron-dependent cell death of hepatocellular carcinoma cells exposed to sorafenib. *International Journal of Cancer*.

[8] Louandre C., Marcq I., Bouhlal H. (2015). The retinoblastoma (Rb) protein regulates ferroptosis induced by sorafenib in human hepatocellular carcinoma cells. *Cancer Letters*.

[9] Eling N., Reuter L., Hazin J., Hamacher-Brady A., Brady N. R. (2015). Identification of artesunate as a specific activator of ferroptosis in pancreatic cancer cells. *Oncoscience*.

[10] Liu Z., Zhao Q., Zuo Z. X. (2020). Systematic analysis of the aberrances and functional implications of ferroptosis in cancer. *Iscience*.

[11] Cancer Genome Atlas Research Network, Brat D. J., Verhaak R. G. W. (2015). Comprehensive, integrative genomic analysis of diffuse lower-grade gliomas. *The New England Journal of Medicine*.

[12] Jiang T., Nam D. H., Ram Z. (2021). Clinical practice guidelines for the management of adult diffuse gliomas. *Cancer Letters*.

[13] Weller M., Wick W., Aldape K. (2015). Glioma. *Nature Reviews. Disease Primers*.

[14] Lim M., Xia Y., Bettegowda C., Weller M. (2018). Current state of immunotherapy for glioblastoma. *Nature Reviews. Clinical Oncology*.

[15] Jiang T., Mao Y., Ma W. (2016). CGCG clinical practice guidelines for the management of adult diffuse gliomas. *Cancer Letters*.

[16] Shibue T., Weinberg R. A. (2017). EMT, CSCs, and drug resistance: the mechanistic link and clinical implications. *Nature Reviews. Clinical Oncology*.

[17] Xu S., Tang L., Li X., Fan F., Liu Z. (2020). Immunotherapy for glioma: current management and future application. *Cancer Letters*.

[18] Wang Z., Su G., Dai Z. (2021). Circadian clock genes promote glioma progression by affecting tumour immune infiltration and tumour cell proliferation. *Cell Proliferation*.

[19] Bloch O., Crane C. A., Kaur R., Safaee M., Rutkowski M. J., Parsa A. T. (2013). Gliomas promote immunosuppression through induction of B7-H1 expression in tumor-associated macrophages. *Clinical Cancer Research*.

[20] Kaminska B., Kocyk M., Kijewska M. (2013). TGF beta signaling and its role in glioma pathogenesis. *Advances in Experimental Medicine and Biology*.

[21] Wainwright D. A., Balyasnikova I. V., Chang A. L. (2012). IDO expression in brain tumors increases the recruitment of regulatory T cells and negatively impacts survival. *Clinical Cancer Research*.

[22] Wagner S., Czub S., Greif M. (1999). Microglial/macrophage expression of interleukin 10 in human glioblastomas. *International Journal of Cancer*.

[23] Oberoi R. K., Parrish K. E., Sio T. T., Mittapalli R. K., Elmquist W. F., Sarkaria J. N. (2016). Strategies to improve delivery of anticancer drugs across the blood-brain barrier to treat glioblastoma. *Neuro-Oncology*.

[24] Ballabh P., Braun A., Nedergaard M. (2004). The blood-brain barrier: an overview: structure, regulation, and clinical implications. *Neurobiology of Disease*.

[25] Du J., Ji H., Ma S. (2021). m6A regulator-mediated methylation modification patterns and characteristics of immunity and stemness in low-grade glioma. *Briefings in Bioinformatics*.

[26] Yang K., Wu Z., Zhang H. (2022). Glioma targeted therapy: insight into future of molecular approaches. *Molecular Cancer*.

[27] Liang X., Wang Z., Dai Z., Zhang H., Cheng Q., Liu Z. (2021). Promoting prognostic model application: a review based on gliomas. *Journal of Oncology*.

[28] Gautier L., Cope L., Bolstad B. M., Irizarry R. A. (2004). Affy--analysis of Affymetrix GeneChip data at the probe level. *Bioinformatics*.

[29] Wilson C. L., Miller C. J. (2005). Simpleaffy: a BioConductor package for Affymetrix quality control and data analysis. *Bioinformatics*.

[30] Leek J. T., Johnson W. E., Parker H. S., Jaffe A. E., Storey J. D. (2012). The sva package for removing batch effects and other unwanted variation in high-throughput experiments. *Bioinformatics*.

[31] Gaujoux R., Seoighe C. (2010). A flexible R package for nonnegative matrix factorization. *BMC Bioinformatics*.

[32] Hanzelmann S., Castelo R., Guinney J. (2013). GSVA: gene set variation analysis for microarray and RNA-seq data. *BMC Bioinformatics*.

[33] Mariathasan S., Turley S. J., Nickles D. (2018). TGF*β* attenuates tumour response to PD-L1 blockade by contributing to exclusion of T cells. *Nature*.

[34] Yu G., Wang L. G., Han Y., He Q. Y. (2012). Clusterprofiler: an R package for comparing biological themes among gene clusters. *OMICS*.

[35] Charoentong P., Finotello F., Angelova M. (2017). Pan-cancer Immunogenomic analyses reveal genotype-immunophenotype relationships and predictors of response to checkpoint blockade. *Cell Reports*.

[36] Jia Q., Wu W., Wang Y. (2018). Local mutational diversity drives intratumoral immune heterogeneity in non- small cell lung cancer. *Nature Communications*.

[37] Newman A. M., Steen C. B., Liu C. L. (2019). Determining cell type abundance and expression from bulk tissues with digital cytometry. *Nature Biotechnology*.

[38] Ritchie M. E., Phipson B., Wu D. (2015). Limma powers differential expression analyses for RNA-sequencing and microarray studies. *Nucleic Acids Research*.

[39] Jiang P., Gu S., Pan D. (2018). Signatures of T cell dysfunction and exclusion predict cancer immunotherapy response. *Nature Medicine*.

[40] Yoshihara K., Shahmoradgoli M., Martinez E. (2013). Inferring tumour purity and stromal and immune cell admixture from expression data. *Nature Communications*.

[41] Necchi A., Joseph R. W., Loriot Y. (2017). Atezolizumab in platinum-treated locally advanced or metastatic urothelial carcinoma: post-progression outcomes from the phase II IMvigor210 study. *Annals of Oncology*.

[42] Riaz N., Havel J. J., Makarov V. (2017). Tumor and microenvironment evolution during immunotherapy with nivolumab. *Cell*.

[43] Gide T. N., Quek C., Menzies A. M. (2019). Distinct immune cell populations define response to anti-PD-1 monotherapy and anti-PD-1/anti-CTLA-4 combined therapy. *Cancer Cell*.

[44] Basu A., Bodycombe N. E., Cheah J. H. (2013). An interactive resource to identify cancer genetic and lineage dependencies targeted by small molecules. *Cell*.

[45] Rees M. G., Seashore-Ludlow B., Cheah J. H. (2016). Correlating chemical sensitivity and basal gene expression reveals mechanism of action. *Nature Chemical Biology*.

[46] Seashore-Ludlow B., Rees M. G., Cheah J. H. (2015). Harnessing connectivity in a large-scale small-molecule sensitivity dataset. *Cancer Discovery*.

[47] Geeleher P., Cox N., Huang R. S. (2014). pRRophetic: an R package for prediction of clinical chemotherapeutic response from tumor gene expression levels. *PLoS One*.

[48] Riquelme I., Tapia O., Espinoza J. A. (2016). The gene expression status of the PI3K/AKT/mTOR pathway in gastric cancer tissues and cell lines. *Pathology Oncology Research*.

[49] Nakaya M., Xiao Y., Zhou X. (2014). Inflammatory T cell responses rely on amino acid transporter ASCT2 facilitation of glutamine uptake and mTORC1 kinase activation. *Immunity*.

[50] Yi J., Zhu J., Wu J., Thompson C. B., Jiang X. (2020). Oncogenic activation of PI3K-AKT-mTOR signaling suppresses ferroptosis via SREBP-mediated lipogenesis. *Proceedings of the National Academy of Sciences of the United States of America*.

[51] Wang Z., Dai Z., Zheng L. (2021). Ferroptosis activation scoring model assists in chemotherapeutic agents’ selection and mediates cross-talk with immunocytes in malignant glioblastoma. *Frontiers in Immunology*.

[52] Wang W., Green M., Choi J. E. (2019). CD8^+^ T cells regulate tumour ferroptosis during cancer immunotherapy. *Nature*.

[53] Lang X., Green M. D., Wang W. (2019). Radiotherapy and immunotherapy promote tumoral lipid oxidation and ferroptosis via synergistic repression of SLC7A11. *Cancer Discovery*.

[54] Tang R., Xu J., Zhang B. (2020). Ferroptosis, necroptosis, and pyroptosis in anticancer immunity. *Journal of Hematology & Oncology*.

[55] Yee P. P., Wei Y., Kim S. Y. (2020). Neutrophil-induced ferroptosis promotes tumor necrosis in glioblastoma progression. *Nature Communications*.

[56] Zeng C., Tang H., Chen H., Li M., Xiong D. (2020). Ferroptosis: a new approach for immunotherapy. *Cell Death Discovery*.

