# Supplementary material for: Transcriptional Patterns of Lower-Grade Glioma Patients with Distinct Ferroptosis Levels, Immunotherapy Response, and Temozolomide Sensitivity
Source: Oxid Med Cell Longev. 2022 May 10;2022:9408886. doi: 10.1155/2022/9408886 (PMC9113876; doi:10.1155/2022/9408886)
Supplement: Supplementary Materials — Figure S1: (A–C) Kaplan-Meier curves revealed the FPI could stratified LGG patients into low- and high-FPI subgroup with distinct prognosis in the TCGA (A, log-rank test, p < 0.0001), meta-CGGA (B, log-rank test, p < 0.0001), and meta-GEO (C, log-rank test, p < 0.048) cohorts. (D) The significant enriched terms of the 24 FRGs in Metascape enrichment analysis. (E–G) The Spearman correlation heat maps showed the correlations between every two FRGs in the TCGA (E), meta-CGGA (F), and meta-GEO (G) cohorts. (H–J) The forest plots showed the prognostic role of the 24 FRGs in the TCGA (H), meta-CGGA (I), and meta-GEO (J) cohorts. The red name represents antiferroptosis regulator, and blue means proferroptosis regulator. Figure S2: (A) The correlation between cophenetic, dispersion, evar, residuals, rss, silhouette, and sparseness coefficients with the number of unsupervised NMF clusters. (B) Heat map of two unsupervised NMF clusters for ferroptosis regulators in the TCGA-LGG cohort. (C–E) Heat maps depict the expression levels of 24 ferroptosis regulator genes in TCGA (C), meta-CGGA (D), and meta-GEO (E) cohorts and annotated with FRCluster, age, gender, and grade information. (F–H) Principal component analysis (PCA) of the FRC clusters based on the whole transcriptional profile in the TCGA (F), meta-CGGA (G), and meta-GEO (H) cohorts. Figure S3: (A) The comparisons of 28 immune cell fractions quantified by ssGSEA algorithm between FRC1 and FRC2 LGG subgroups (Wilcoxon rank-sum test). (B) The comparisons of CIBERSORT immune cell fractions between FRC1 and FRC2 LGG subgroups (Wilcoxon rank-sum test). (C–E) Neoantigen (C), LOH fractions (D), and stemness index (E) were compared between FRC1 and FRC2 LGG subgroups (Wilcoxon rank-sum test). The labelled asterisk indicated the statistical p value (ns p > 0.05, ∗p < 0.05, ∗∗p < 0.01, and ∗∗∗p < 0.001). Figure S4: (A, B) Kaplan-Meier curves for low- and high-FRscore LGG subgroups in the meta-CGGA cohort (A) and meta-GEO cohort (B [file 9408886.f1.pdf]

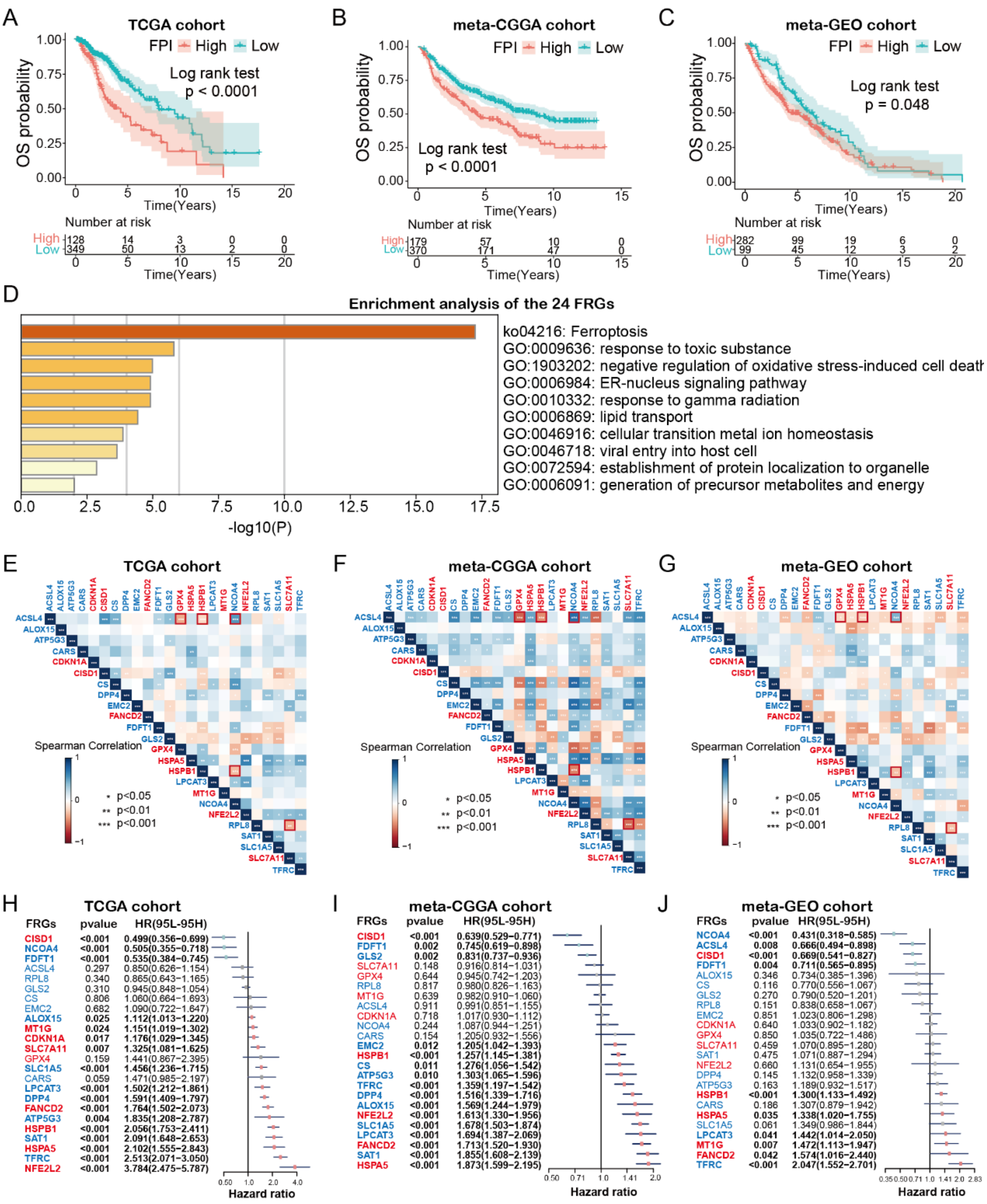

Figure S1. (A-C) Kaplan-Meier curves revealed the FPI could stratified LGG patients into low- and high-FPI subgroup with distinct prognosis in the TCGA (A, log-rank test, p<0.0001), meta-CGGA (B, log-rank test, p<0.0001) and meta-GEO (C, log-rank test, p<0.048) cohorts. **(D)** The significant enriched terms of the 24 FRGs in Metascape enrichment analysis. **(E-G)** The spearman correlation heatmaps showed the correlations between every two FRGs in the TCGA (E), meta-CGGA (F) and meta-GEO (G) cohorts. **(H-J)** The forest plots showed the prognostic role of the 24 FRGs in the TCGA (H), meta-CGGA (I) and meta-GEO (J) cohorts. The red name represents anti-ferroptosis regulator and blue means pro-ferroptosis regulator.

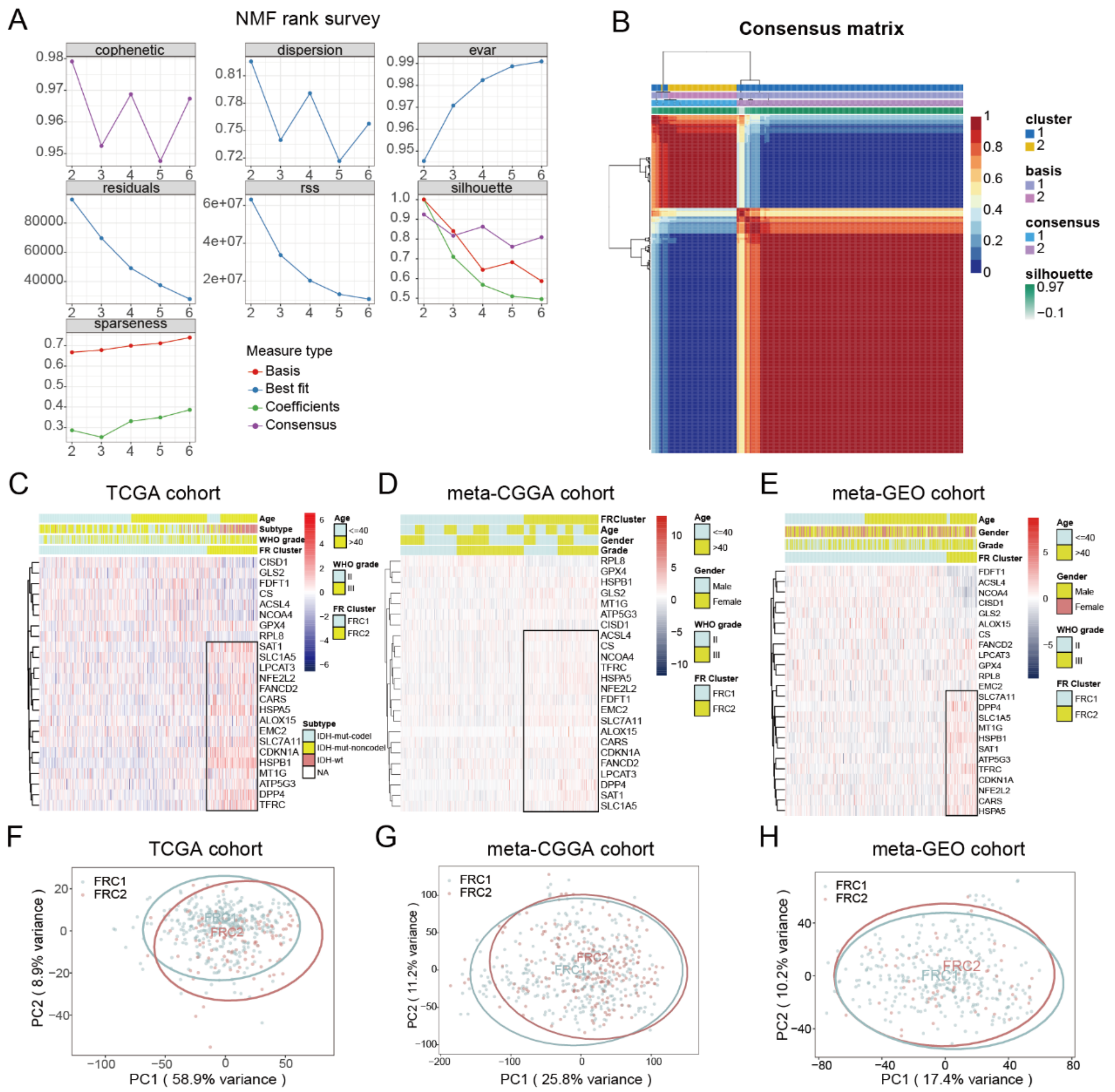

Figure S2. (A) The correlation between cophenetic, dispersion, evar, residuals, rss, silhouette, and sparseness coefficients with the number of unsupervised NMF clusters. **(B)** Heatmap of two unsupervised NMF clusters for ferroptosis regulators in the TCGA-LGG cohort. **(C-E)** Heatmaps depict the expression levels of 24 ferroptosis regulator genes in TCGA (C), meta-CGGA (D) and meta-GEO (E) cohorts, and annotated with FRCluster, age, gender and grade information. **(F-H)** Principal component analysis (PCA) of the FRC clusters based on the whole transcriptional profile in the TCGA (F), meta-CGGA (G) and meta-GEO (H) cohorts.

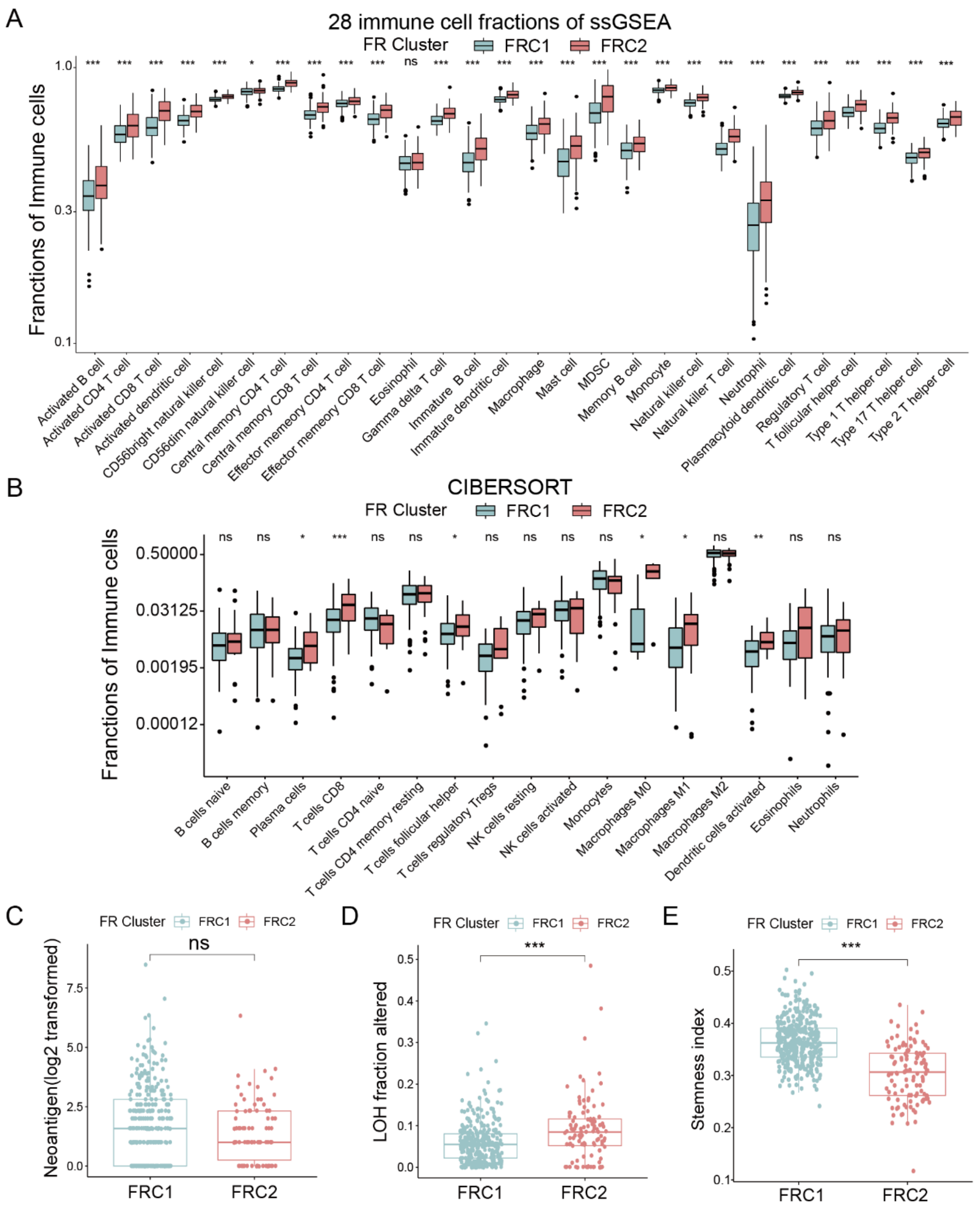

Figure S3. (A) The comparisons of 28 immune cell fractions quantified by ssGSEA algorithm between FRC1 and FRC2 LGG subgroups (Wilcoxon rank-sum test). **(B)** The comparisons of CIBERSORT immune cell fractions between FRC1 and FRC2 LGG subgroups. (Wilcoxon rank-sum test) **(C-E)** Neoantigen (C), LOH fractions (D) and stemness index (E) were compared between FRC1 and FRC2 LGG subgroups. (Wilcoxon rank-sum test). The labelled asterisk indicated the statistical p value (ns p>0.05, *p<0.05, **p<0.01 and ***p<0.001).

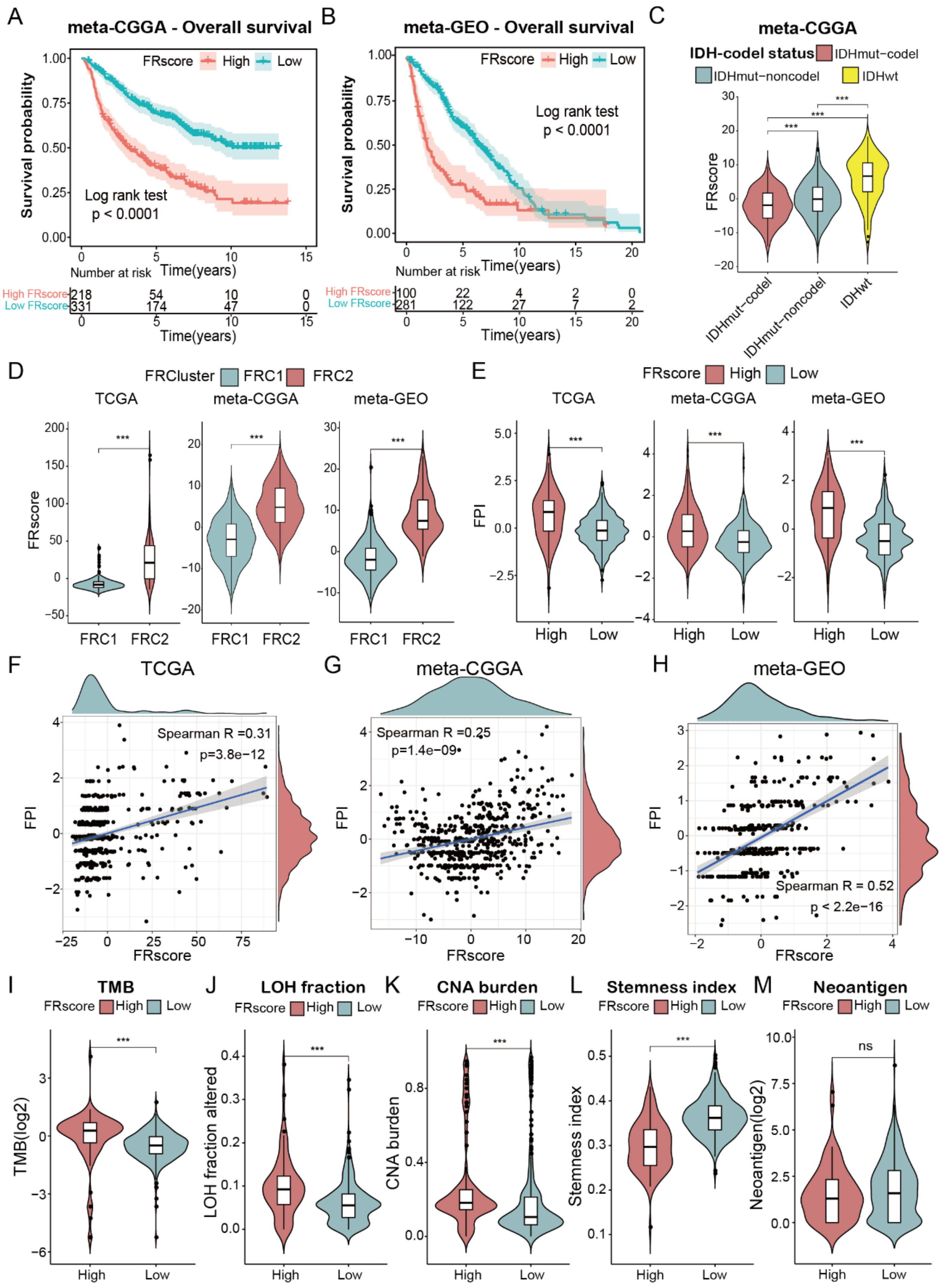

Figure S4. (A-B) Kaplan-Meier curves for low- and high-FRscore LGG subgroups in the meta-CGGA cohort

(A) and meta-GEO cohort (B) present LGG patients with higher FRscores have poorer prognosis. **(C)** Distribution of FRscore in distinct IDH-codeletion subtypes in the meta-CGGA cohort, and the FRscore levels between every two molecular subgroups were compared by Wilcoxon rank-sum test. **(D)** Comparison of FRscores between FRC1 and FRC2 LGG subgroups in the three LGG cohorts. (Wilcoxon rank-sum test). **(E)** Comparison of FPI levels between FRC1 and FRC2 LGG subgroups in the three LGG cohorts. (Wilcoxon rank-sum test). **(F-H)** Spearman correlation analysis showed the positive correlation between FPI and FRscore in the three LGG cohort. **(I-M)** Tumor mutation burden (TMB, I), loss of heterozygosity (LOH) fraction (J), copy number alteration (CNA) burden (K), stemness index (L) and neoantigen (M) levels were compared between low- and high-FRscore LGG subgroups. (Wilcoxon rank-sum test). The labelled asterisk indicated the statistical p value (ns $p>0.05$ and ***$p<0.001$).
